# Navicular Disease (Navicular Arthritis)1Read before the Virginia State Veterinary Medical Association, June 24, 1896.

**Published:** 1896-09

**Authors:** H. L. Drake


### NAVICULAR DISEASE (NAVICULAR ARTHRITIS).[1]

By H. L. DRAKE, D.V.S.

THE veterinary profession is indebted to Mr. James Turner for a knowledge of the seat and cause of the disease known as navicular arthritis. He alludes to it first in the year 1816, and though eighty years have rolled away little has been done since his day revealing new pathological or even etiological changes or discoveries.

*Etiology.* We comprehend more fully how navicular disease may be caused when we recall the peculiar anatomy of the parts involved in the process and the function that is performed by locomotion. The anterior extremities have the bulk of the body-weight to bear, and their support is by elastic-sling mus-

[1] Read before the Virginia State Veterinary Medical Association, June 24, 1896.

cles which bind the shoulder to the body, thus greatly dissipating the shock to the foot. Then the foot is composed of elastic material throughout, protecting the sensitive structure; but even this is not sufficient of itself to protect the foot from injury, so nature has further provided for exigency by placing the coffin-joint on the posterior part of the coffin-bone, instead of directly on top of it, whereby a large part of the shock of locomotion is dispersed before it can reach the vertical column represented in the common knee and arm bones. In addition to these provisions the frog, plantar cushion, flexor tendons, interarticular cartilages, and connective tissue are interposed to break the concussion and shock.

Such are the means provided for dispelling the shock to the joints and vertical bones, and, ample as they seem to appear, they nevertheless fail to relieve the parts from concussion and excessive pressure of the sensitive structure within the hoof, due to rapid pounding over hard surfaces. Hence there arises a bruise of the navicular bone, the bursa in connection therewith, and the perforans tendon; and the disease is chiefly found in those animals that are high-knee actors or rapid pacemakers.

Professors Smith and Law differ as to the class of horses among which it is most commonly found. Smith says: "It is rare in racing, hunting, and even in trotting horses, as long as they are used on the turf." Law says: "The thoroughbred is more commonly affected with the disease than any other." But the fact is that besides the exciting causes must be considered the predisposition in individual animals, most prominent among which are heredity and vice of conformation, and added to this the environments of domestication and use, such as dry stables, *i. e.*, board floors in stables, hard and fast work, bad shoeing, and punctured wounds.

Mr. Turner writes: "Contraction mostly takes place in animals that have been accustomed to be shod before the age at which they have attained their highest value for work, viz., five years, but this contraction is not, however, necessarily connected with lameness. The next deviation from nature is the passive state to which the foot is subjected for twenty-four hours, and sometimes several days. Compare this with the few hours during which a horse in a state of nature, that is, roaming at will in the pasture-field, is in a quiescent condition, and there will be no cause for surprise in the change of form, position, character, and the state of contraction which takes place in the foot deprived of its natural pressure and motion.

The first consequence of contraction is the gradual displacement of the navicular and os pedis bones. They ascend within the hoof, and an unnatural arch is formed by the ascent of the frog.

The delicate synovial membranes lining the joint are crushed and bruised by the very material which nature has bestowed as a defence. We learn from this writer that the bruising of the synovial membrane lining this joint is the veritable source of this disease. It is engendered in the stable, but becomes permanently established by sudden violence out of it. Horses doing fast work on our hard roads, and those subject to the whims of fashion by being trained to high knee-action, are the ones that suffer most from this dreaded disease.

From the time of its discovery Turner shows conclusively that he was acquainted with the cause of navicular disease and its pathological changes.

*Symptoms.* In the early stage the symptoms are obscure. The animals may be taken suddenly lame for a few days, and then the lameness may entirely disappear for a short period, only to reappear in a more violent form, which finally becomes constant and aggravated. In the primary stage there may be heat about the base and posterior part of the foot; but in some instances there is no perceptible change in the temperature about the parts. The most characteristic symptoms are pointing of the affected foot when at rest, and clean appearance of limb, due to atrophy of muscles from imperfect use.

This may be seen before the lameness is manifested. The affected foot always takes a short step and the toe of the foot first strikes the ground, so that the shoe is most worn at that point. The animal has a stumbling gait, and when both feet are affected at the same time shows a stilty movement, as though he had both legs tied closely together. When made to work shows much pain and sweats profusely; also shows much pain on pressure over the seat of disease. There never was yet a philosopher who could withstand a toothache; but think of a poor horse with twenty toothaches compressed into one agony. The leg is bent with difficulty and pain, owing to the flexion of the perforans tendons upon the navicular bone.

The course of this disease is usually inflammatory, followed by ulceration, necrosis, or ossification of bones of coffin-joint. If the disease is of long standing, there is marked contraction of horny substance of foot.

*Prognosis.* Prognosis of this disease is very unfavorable in cases of long standing, owing to its destructiveness to the joint and its membranes involved by this disease.

*Post-mortem.* Numerous dissections have shown that the navicular bone and tendons forming that joint are invariably the seat of this obscure disease. The cartilage of this joint has been found in an ulcerated condition with the bursal membrane destroyed, the bone showing caries in bad cases, and in others bony adhesions have taken place in the joint formed by the navicular bone and the os pedis, and in a few cases the foot has become disorganized and useless by ravages of this terrible disease.

*Treatment.* Nothing has yet been found by the profession to cure an established case of navicular disease if disorganization has taken place, although many would have us believe that they have the sure and only cure. But in cases where a cure has been effected it is certain that disorganization has not occurred in the parts affected.

We have a number of palliative remedies that are in vogue to-day, of which neurotomy stands at the head, and is mostly resorted to by the profession in city practice. The common remedy to relieve the suffering animals is to pare the foot well down, rasping the walls at heels thin, and place in a tub of medicated water for a few hours. Soak each day until a new growth is established. This may be carried too far, making the foot spongy, which must be avoided.

The soaking is followed by a good blister around the coronary substance; after the action of the blister has subsided, placing an expanding spring in angles of the heel and shoeing with light tips, and turning out on a meadow bottom for a period of rest, is a common treatment.

It only remains to be said that in all regulated stables care should be taken to keep the horse standing with his front feet resting on clay, or, in absence of clay, to stuff the feet periodically with flaxseed poultices or clay mixed with vinegar; to give regular exercise, and to avoid severe usage on hard roads; and frequent careful shoeing.

